# The Predictive Value of Estrogen Receptor 1 on Adjuvant Chemotherapy in Locally Advanced Colorectal Cancer: A Retrospective Analysis With Independent Validation and Its Potential Mechanism

**DOI:** 10.3389/fonc.2020.00214

**Published:** 2020-03-20

**Authors:** Shu-Biao Ye, Yi-Kan Cheng, Ru Deng, Yanhong Deng, Peisi Li, Lin Zhang, Ping Lan

**Affiliations:** ^1^Guangdong Provincial Key Laboratory of Colorectal and Pelvic Floor Diseases, Department of Colorectal Surgery, Guangdong Institute of Gastroenterology, The Sixth Affiliated Hospital, Sun Yat-sen University, Guangzhou, China; ^2^Guangdong Provincial Key Laboratory of Colorectal and Pelvic Floor Diseases, Department of Radiation Oncology, Guangdong Institute of Gastroenterology, Guangzhou, China; ^3^Guangdong Provincial Key Laboratory of Colorectal and Pelvic Floor Diseases, Department of Oncology, Guangdong Institute of Gastroenterology, The Sixth Affiliated Hospital, Sun Yat-sen University, Guangzhou, China; ^4^School of Medicine, Sun Yat-sen University, Guangzhou, China; ^5^State Key Laboratory of Oncology in South China, Collaborative Innovation Center for Cancer Medicine, Department of Clinical Laboratory, Sun Yat-sen University Cancer Center, Guangzhou, China

**Keywords:** colorectal cancer, ESR1, predictive, adjuvant chemotherapy, retrospective analysis

## Abstract

**Purpose:** To investigate the predictive biomarker value of estrogen receptor 1 (ESR1) expression in tumor tissue on adjuvant chemotherapy in curatively resected colorectal cancer (CRC).

**Methods:** A total of 467 CRC patients in 2007–2010 were retrospectively evaluated. Clinical information and follow-up data were retrieved from hospital registries and patient files. What's more, we used an external independent cohort (*n* = 511) from GSE39582 for further validation. Overall survival was estimated by the Kaplan–Meier method, and the survival curves were compared by log-rank tests. Cox proportional hazards models were used for multivariate analyses to calculate the hazard ratios (HRs) and test independent significance. Immunohistochemistry and Western blot were applied to detect protein expression of ESR1 in CRC patients and cell lines. The stable knockdown and overexpressed cells were transduced with the lentivirus. Cell viability was measured by an MTS reagent.

**Results:** The predictive value of ESR1 was investigated in locally advanced CRC patients. Kaplan–Meier analysis indicated that ESR1 expression was significantly correlated with OS in patients receiving adjuvant chemotherapy from these cohorts, with *p* = 0.015 and *p* < 0.001, respectively. ESR1 expression was significantly correlated with 5-flurouracil (5-FU)-based adjuvant chemotherapy in training with an HR of 1.792 (95%CI: 1.100–2.921, *p* = 0.019). Downregulation of ESR1 was related with enhanced chemosensitivity to 5-FU in CRC cell lines, while upregulation of ESR1 was correlated with decreased chemosensitivity.

**Conclusions:** The present study manifest clinical validity of ESR1 expression as a predictive biomarker on 5-FU-based adjuvant chemotherapy in stage II–III CRC.

## Introduction

Colorectal cancer (CRC) incidence in China has been increasing over the past decades (1–3). CRC ranks fifth in the causes of cancer morbidity and mortality (4, 5). 5-Flurouracil (5-FU)-based adjuvant chemotherapy is the standard treatment for locally advanced CRC patients. However, up to 40% of advanced CRC patients cannot benefit from adjuvant chemotherapy and will eventually relapse (6).

Great effort has been made to identify markers to predict the benefit from adjuvant chemotherapy. However, the only biomarker at present in clinical use is carcinoembryonic antigen (CEA), which is widely used to monitor treatment response and to detect recurrence (7). A specimen-derived biomarker from surgery is urgently required to determine the following adjuvant treatment. Presently the treatment is mainly based on the tumor/node/metastasis (TNM) stage. Therefore, it is quite crucial to identify and validate markers from surgical pathologic samples, which enables identification of patients at high and low risk of early relapse and progression, independently of the tumor stage. Thus, the biomarkers have great implication in individualized adjuvant treatment and follow-up. The primary aim of the present study was to evaluate the tumor expression of estrogen receptor 1 (ESR1) from CRC patients in this context.

ESR1, a nuclear receptor subfamily 3, group A, member 1, is one of two main types of estrogen receptor (ER), which is activated by the sex hormone estrogen. Although ESR1 expression levels remain low in both normal and cancerous colonocytes, our recent study revealed that ESR1 expression attributed to inferior clinical outcome in CRC patients (8). Moreover, other studies indicated that ESR1 may be implicated in the development and progression of CRCs (9–11). However, whether ESR1 expression can predict the efficacy of adjuvant chemotherapy to influence survival and how it regulates chemosensitivity remain unclear. The primary objective of this study was therefore to explore the correlation between ESR1 expression and adjuvant chemotherapy outcome in training and validation cohorts of locally advanced CRC patients.

## Materials and Methods

### Clinical Specimens

We retrospectively collected 467 paraffin-embedded resection samples from patients from November 2007 until October 2010. Those eligible for inclusion were patients who underwent curative resection for stage II–III CRC at Sun Yat-Sen University Cancer Center (South China). All biological experiments were conducted under the “Laboratory Safety Rules of Guangdong Institute of Gastroenterology” thus, standard biosecurity and institutional safety procedures have been adhered to Standardized Operation. Patients receiving neoadjuvant treatment were excluded. Clinical characteristics including age, gender, tumor localization, tumor stage, adjuvant chemotherapy, chemotherapy regimen, and survival were obtained from patient files and registries.

### Treatment

The treatment strategy was determined by clinical stage, the multidisciplinary team's (MDT) decision, and patient choice. All patients from training cohort received definitive-intent surgery. Patients have not received adjuvant chemotherapy mainly due to patients' refusal. 5-FU-based chemotherapy was delivered. Adjuvant chemotherapy consisted of oxaliplatin (130 mg/m^2^, day 1) with capecitabine (1,000 mg/m^2^, bid, days 1–14) or 5-FU (bolus 400 mg/m^2^ and then 1,200 mg/m^2^/day over 46–48 h) every 3 weeks or Leucovorin (400 or 200 mg/m^2^, day 1), with 5-FU (bolus 400 mg/m^2^ and then 1,200 mg/m^2^/day over 46–48 h) every 2 weeks.

### Immunohistochemistry

Firstly, the fresh tissues were fixed in 10% buffered formalin (PH 7.0) and embedded in paraffin. The paraffin-embedded tumor samples were then sectioned continuously into 4-μm-thick sections. Then the sections were dewaxed in xylene and rehydrated in graded alcohols. A normal rabbit IgG antibody was used as a negative control. Following antigen retrieval by microwave heating (95°C for 20 min), sections were then incubated with ESR1 (Clone SP1, Ventana Medical Systems. Inc.) at 4°C overnight. After washing, a horseradish peroxidase-labeled goat antibody against a mouse/rabbit secondary antibody (Envision; Dako, Glostrup, Denmark) was added, followed by incubation at room temperature for 30 min. The signal was then detected with 3,3′-diaminobenzidine tetrahydrochloride (DAB). Two independent pathologists blinded to the clinical information scored the ESR1 expression levels in tumor cells by assessing (a) the proportion of positively stained cells (0, < 5%; 1, 6–25%; 2, 26–50%; 3, 51–75%; 4, >75%) and (b) the signal intensity (0, no signal; 1, weak; 2, moderate; 3, strong). The score was the product of a × b. Considering that the number of patients with score > 0 in ESR1 was very low, we used dichotomic classification (positive/negative). Therefore, a positive group (a × b>0) and a negative group (a × b = 0) were used (Figure S1).

### Gene Expression Analyses

Two public data sets were analyzed in the validation stage, CRC mRNA microarray data GSE39582 and GSE14333 from Gene Expression Omnibus (GEO). Preprocessed data from GEO were downloaded using the Bioconductor package “GEO query.” The prognosis performance of ESR1 was assessed with Kaplan–Meier survival analysis.

### Cell Growth Inhibition Assay

HCT116, KM12, DLD1, and SW480 cell lines were the cell lines that we used in our study. These cell lines are widely recognized CRC cell lines for research. And more importantly, we have selected two cell lines (DLD1, SW480) with high ESR1 expression and another two cell lines (HCT116, KM12) with low ESR1 expression for further research. The cells were resuspended in the medium as single-cell suspension and seeded in a 96-well plate at a density of 5 × 103 cells/well. After culture overnight, 5-FU was serially diluted with a culture medium and added in a 96-well plate. After incubation for 48 h, 20 μl of MTS reagent (G358A, Promega) was pipetted into each well of the 96-well plate and incubated for 4 h in a 37°C incubator. The absorbance was determined by a microplate analyzer OD490 at 490 nm. Cell survival rate = (OD490 experimental hole -OD490 blank hole)/(OD490 control hole -OD490 blank hole) 100%. Experiments were performed in triplicate and the average value was taken.

### Protein Extraction and Western Blotting

Total proteins were lysed by incubating with RIPA buffer (Beyotime, China) that was supplemented with a protease inhibitor cocktail (Sigma, China). The protein concentrations were measured using a BCA Protein Assay Kit (Boster, China). Proteins were separated by SDS-PAGE using a 10% polyacrylamide gel and transferred to nitrocellulose membranes (BioRad). After blocking by 5% milk for 1 h at room temperature, the membranes were incubated with a relative antibody at 4°C overnight. Membranes were incubated with HRP-conjugated goat anti-mouse antibody (1:10,000) for 1 h. The signal strength of revealed protein bands was detected with an enhanced chemiluminescence (ECL) substrate and captured with Image-Lab software (BioRad). Detection of the relative protein band intensities of GAPDH served as an internal loading control.

### The Establishment of Stable Knockdown and Overexpressed Cells

The open-reading frame of ESR1 was amplified from the cDNA of 293T cells and inserted into the Psin vector. CRC cells were stably transduced with ESR1 overexpressing lentiviral and Psin control vectors. For the ESR1 knockdown cells, CRC cells were transfected with PLKO vectors and transduced with ESR1 knockdown lentiviral vectors (ESR1 sh01: GGAGAATGTTGAAACACAA, sh02: CCAGTGCACCATTGATAAA). Three Lentiviral Packaging Plasmid (psPAX2: pMD2.G = 6: 3: 1.5 μg) were used to transfecting into 293T cells. The released virus was harvested and concentrated by ultracentrifugation. To establish the stable knockdown and overexpressing cells, all cells were transduced with the lentivirus at a multiplicity of infection (MOI) of 10 with 5 μg/ml polybrene (Sigma). The stable expressing cells were selected by treating with puromycin for 2 weeks.

### Statistical Analysis

Statistical analyses were performed in SPSS version 19 (IBM, Corporation, Armonk, NY, USA) and R 3.2.4. Software (http://www.r-project.org). Patient characteristics were compared by *t*-test and the χ^2^ test of independence. Overall survival was estimated by the Kaplan–Meier method, and the survival curves were compared by log-rank tests. The optimal cutoff value of the ESR1 expression level was determined using the X-tile (Yale University, New Haven, CT, USA) software (12). OS was measured from commencement of treatment to death or the date of last follow-up visit for surviving patients. The median follow-up duration was 46.7 months for training. The main causes of death were relapse of tumor and cardiovascular and cerebrovascular diseases.

Cox proportional hazards models were used for multivariate analyses to calculate the hazard ratios (HRs) and to test independent significance. A test for an interaction between adjuvant chemotherapy and ESR1 expression was pre-specified. The training set has developed a model for survival, whose variables were then applied in the validation set. The chosen variables were already well-established clinically relevant covariates such as demographic parameters (age, gender), disease stage, localization, and adjuvant treatment. The criterion for statistical significance was set at p = 0.05 on two-sided tests.

## Results

### ESR1 Expression Is Negatively Correlated With Better Overall Survival (OS) in Locally Advanced Patients Treated With Adjuvant Chemotherapy in the Training Set

To investigate the clinical relevance of ESR1 expression, the present study analyzed ESR1 expression in tumor tissue from 467 locally advanced CRC patients by IHC staining. Among these patients, 337 patients were treated with adjuvant chemotherapy. Seventy-two (32%) stage II patients and 58 (24%) stage III patients did not receive adjuvant chemotherapy. The rate of ESR1 expression was significantly higher in colon tumor tissue compared with that in rectum (*p* = 0.002). The correlation between ESR1 and tumor location from GSE39582 dataset was further validated (Table 1).

**Table 1:** Correlation between expression of ESR1 and clinicopathological features in training and validation sets.

**Factors**	**Training set**		***p***	**Validation set**		***p***
	**ESR1 expression**			**ESR1 expression**		
	**Positive**	**Negative**		**High**	**Low**	
	73	394		48	412	
**Age**			0.524			1
≤ 60	36	212		13	109	
>60	37	182		35	303	
**Sex**			1.000			0.048
Male	43	233		20	238	
Female	30	161		28	174	
Tumor location^*^			0.002			0.01
Colon/Proximal colon	46	170		28	159	
Rectum/Distal colon	27	224		20	253	
**Stage**			1.000			1
Stage II	35	190		27	232	
Stage III	38	204		21	180	
**Adjuvant chemotherapy**			0.67			0.456
Yes	51	286		24	178	
No	22	108		24	234	

*Baseline characteristics of the two cohorts regarding demography (age and gender), tumor features (stage and localization), and ESR1 expression and whether or not patients in stages II–III received adjuvant chemotherapy*.

* *Tumor location for colon cancer patients from external validation cohort was proximal and distal colon*.

To further determine the effect of ESR1 expression on adjuvant chemotherapy in stage II and III CRC patients, the Cox regression model was employed to show strong interactions between ESR1 expression and adjuvant chemotherapy in the multivariate analyses, with *p* = 0.019 (Table 2).

**Table 2:** Multivariate survival analyses including adjuvant chemotherapy on stage II–III CRC patients.

	**Hazard ratio (95% CI)**	***P*-value**
**Number of patients**	467	
**Age**		
>60 vs. ≤ 60 years	0.988 (0.676–1.445)	0.952
**Gender**		
Female vs. male	0.975 (0.664–1.423)	0.896
**Stage**		
Stage III vs. II	1.889 (1.270–2.808)	0.002
**Localization**		
Rectum vs. colon	1.374 (0.935–2.019)	0.106
ESR1^*^ adjuvant chemotherapy	1.792 (1.100–2.921)	0.019

*Multivariate analyses for patients in stages II and III. The variables include age, gender, stage, localization, interaction between ESR1 expression, and adjuvant chemotherapy*.

* *Refers to the interaction between ESR1 expression and adjuvant chemotherapy*.

**Figure 1:**
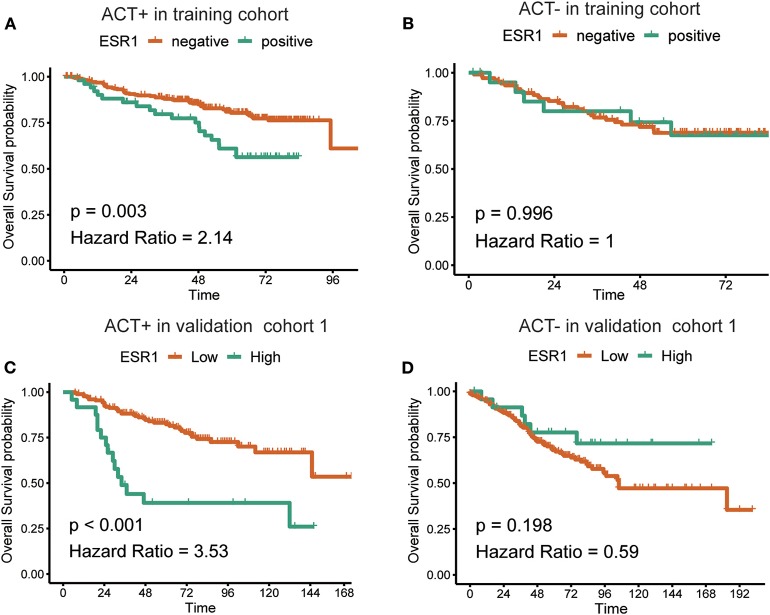
Correlation between the expression of ESR1 and overall survival. Kaplan–Meier survival curves of the overall survival in CRC patients with **(A)** or without **(B)** adjuvant chemotherapy in the training cohort and with **(C)** or without **(D)** adjuvant chemotherapy in the validation cohort. The log-rank analysis was used to test for significance.

**Figure 2:**
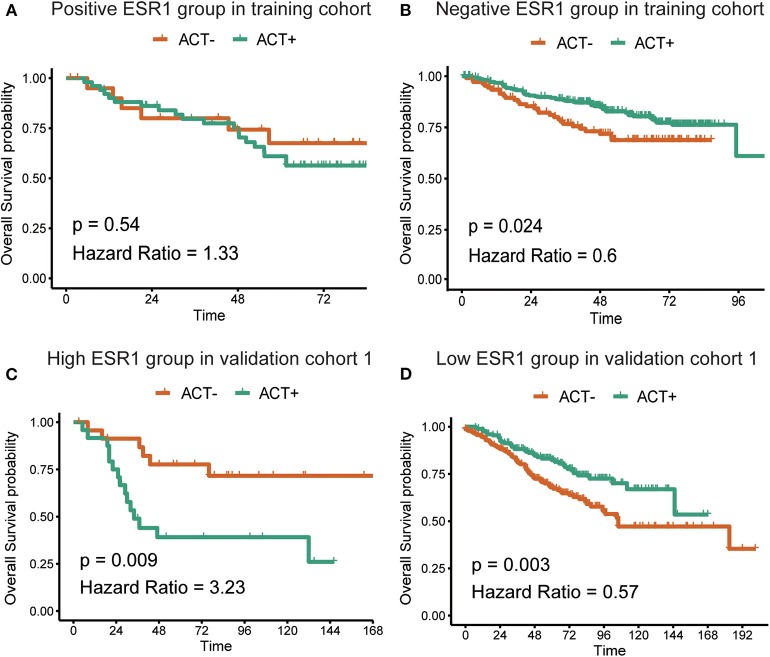
Correlation between adjuvant chemotherapy and overall survival. Kaplan–Meier survival curves of the overall survival in CRC patients with **(A)** or without **(B)** ESR1 expression in the training cohort and with high **(C)** or low **(D)** ESR1 expression in the validation cohort. The log-rank analysis was used to test for significance.

In the additional subgroup analyses, significantly improved overall survival in patients receiving 5-FU-based adjuvant chemotherapy in the training set (*p* = 0.003) and the validation set (*p* < 0.001) was found to be inversely associated with the expression of ESR1 (Figure 1). On the contrary, such association in patients receiving no adjuvant chemotherapy was found to be not significant (Figure 1). For patients without/low ESR1 expression, adjuvant chemotherapy improved overall survival in training (*p* = 0.024) and validation (*p* = 0.024) cohorts comparing with no adjuvant chemotherapy. For patients with ESR1 expression, no correlation between adjuvant chemotherapy and overall survival was found in the training cohort, while adjuvant chemotherapy decreased overall survival in the validation cohort for patients with high ESR1 expression (Figure 2). Moreover, significant inverse association between ESR1 and disease-free survival was found in two independent public datasets (*p* = 0.035 and *p* = 0.006, respectively). Disease-free survival was not associated with ESR1 expression in no adjuvant chemotherapy subgroup (Figure S2).

### ESR1 Expression Is Negatively Associated With CRC Cell Sensitivity to 5-FU

**Figure 3:**
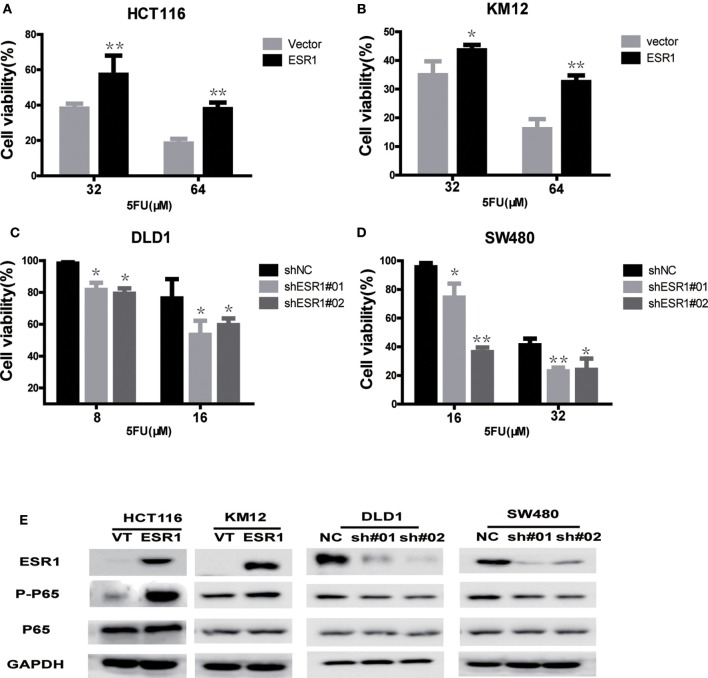
ESR1 expression is negatively correlated with CRC cell sensitivity to 5-FU. ESR1 was up-regulated in **(A)** HCT116 and **(B)** KM12 cells, which were treated with increasing concentrations of 5-FU after transfection with ESR1 or control. Overexpression ESR1 decreases sensitivity to 5-FU. ESR1 was knocked down in **(C)** DLD1 cell and **(D)** sw480 cell, which were treated with increasing concentrations of 5-FU after transfection with ESR1 or control. Knocking down ESR1 gives rise to higher sensitivity to 5-FU. **(E)** KM12 and ESR1 overexpressing HCT116 and KM12 cells displayed increased activation of P65 and ESR1 knocking down DLD1 and sw480 cells displayed decreased activation of P65. *p < 0.05, **p < 0.01.

To further investigate whether the ESR1 level was correlated with CRC cell chemosensitivity to 5-FU *in vitro*, four CRC cell lines were treated with different concentration of 5-FU for 72 h. ESR1-overexpressed cells were less sensitive to 5-FU than control, which led to higher cell viability (Figures 3A,B). Knockdown of ESR1 expression in DLD1 and SW480 cells showed enhancement of cytotoxicity of 5-FU after 72 h of exposure (Figures 3C,D). Thus, these results suggested that ESR1 expression was negatively correlated with CRC cell sensitivity to 5-FU. Western blot analysis indicated that ESR1 overexpressing in CRC cells displayed increased activation of P65 and ESR1 knocking down displayed decreased activation of P65 (Figure 3E).

## Discussion

To the best of our knowledge, this is the first study to investigate the potential predictive role of ESR1 expression in stage II–III CRC patients. This study shows that strong interactions between ESR1 expression and adjuvant chemotherapy existed in both the training set and the validation set. With the use of the validation cohort, this paper reaches evidence II level as defined by Simon et al. (13).

There is a great need for biomarkers from surgical specimen to identify patients with high risk of relapse and progression, thereby allowing for more intensive adjuvant treatment and stricter follow-up with the aim to improve the survival.

The prognostic significance of the biomarker was present in the subpopulation of locally advanced CRC patients receiving adjuvant chemotherapy in the training and validation sets. Patients with low ESR1 expression who accounts for the most majority of CRC patients benefitted from adjuvant chemotherapy in both cohorts. Additionally, our finding may suggest that ESR1 expression in tumor tissue after curative resection is a predictor of poor response to 5-FU-based adjuvant chemotherapy. This indicates that ESR1 may deregulate chemotherapy drugs' sensitivity as it did in breast cancer (14, 15). The exact mechanism behind the drug resistance of ESR1 expression in CRC is largely unknown. The NF-κB pathway was a well-known pathway, which has been reported to be linked to various cellular processes in cancer including chemoresistance (16). P65 is the key protein of the NF-κB pathway, and its phosphorylation activates the NF-κB pathway. Our study indicated that activating phosphorylation of P65 by ESR1 may play a role in the 5-FU resistance, which gave a hint to investigate NF-κB signal pathways. In accordance with our study, NF-κB signal pathways were reported to contribute to chemotherapy resistance in many cancers (17, 18). Furthermore, a recent study also demonstrated that NF-κB played a role in irinotecan-resistant colon cancer cells (19). Therefore, further study to investigate the specific molecular mechanism behind the drug resistance of ESR1 expression is warranted. The significant interaction regarding adjuvant chemotherapy and ESR1 expression in these cohorts indicates that the current adjuvant chemotherapy may not be enough for patients with ESR1 expression. Hormone therapy (HT) remains one of the mainstay treatments for breast cancer patients expressing ESR1. Furthermore, several studies suggested that HT may play a crucial role in its protective effects on colon cancer (20, 21). Although the addition of HT to adjuvant chemotherapy may be a potential strategy for stage II and III patients with ESR1 expression, we would not advocate altering current treatment decisions according to our findings unless it was validated by further clinical trials. This special aspect of the applicability of the biomarker must be further assessed due to the lack of prospective evidence of clinical trials and the increased rates of cardiovascular events with HT (22).

A single biomarker is not possibly sufficient to describe the complex biological characteristics of the tumor and its microenvironment. Sargent et al. showed that patients with dMMR status cannot benefit from 5-FU adjuvant chemotherapy in stage II colon cancer (23), which indicated that MMR status was quite crucial. Although our study indicated the predictive value of ESR1 in stage II–III CRC, further prospective study that validates the predictive value of ESR1 according to the MMR status in CRC is demanded. A series of valid biomarkers of cancer (24) could prove useful for patient-tailored individualized treatments based on molecular features in addition to already established clinicopathological characteristics.

Although the potential biological reason why ESR1 expression affects the prognosis of stage II–III CRC patients receiving adjuvant chemotherapy remains to be specifically clarified, the results validated in the present study indicate the clinical implication of ESR1 expression in these subsets of CRC patients. When treating CRC patients, whether implementation of the ESR1 IHC staining to the clinical routine could offer an improved survival needs prospective studies to evaluate. Interpreting the possibility of the addition of HT to adjuvant chemotherapy in patients with ESR1 expression, this strategy needs to be validated beforehand in CRC cell lines, animal models, and then clinical trials.

## Conclusion

The results of the current study indicated independent predictive influence of tumor cell expression of ESR1 on 5-FU-based adjuvant chemotherapy from stage II–III CRC patients. Additionally, activating phosphorylation of P65 by ESR1 may play a role in the 5-FU resistance. Further sufficiently powered, prospective studies must be performed with concentration on conforming the predictive value.

## Data Availability Statement

The datasets analyzed in this study can be found in the NCBI Gene Expression Omnibus (GSE39582, GSE14333).

## Ethics Statement

This study was approved by the institutional review board of the Sixth Affiliated Hospital, Sun Yat-sen University. Informed consent for research use was waived.

## Author Contributions

S-BY and Y-KC contributed to study design and to writing of the manuscript, performed experiments, and analyzed and interpreted the data. RD performed experiments and analyzed and interpreted the data. PLi and RD performed the experiments. YD contributed to the study design. PLa and LZ conceived and designed the study, analyzed and interpreted the data, and wrote the manuscript. All authors revised the manuscript and approved the final version for submission.

### Conflict of Interest

The authors declare that the research was conducted in the absence of any commercial or financial relationships that could be construed as a potential conflict of interest.

## References

[1] Siegel RL Miller KD Jemal A Cancer statistics 2017. CA Cancer J Clin. (2017) 67:7–30. 10.3322/caac.21387 28055103

[2] Bray F Ferlay J Soerjomataram I Siegel RL Torre LA Jemal A. Global cancer statistics 2018: GLOBOCAN estimates of incidence and mortality worldwide for 36 cancers in 185 countries. CA Cancer J Clin. (2018) 68:394–424. 10.3322/caac.21492 30207593

[3] Chen W Zheng R Baade PD Zhang S Zeng H Bray F. Cancer statistics in China, 2015. CA Cancer J Clin. (2016) 66:115–32. 10.3322/caac.21338 26808342

[4] Chen W Zheng R Zeng H Zhang S He J. Annual report on status of cancer in China, 2011. Chin J Cancer Res. (2015) 27:2–12. 10.3978/j.issn.1000-9604.2015.01.06 25717220 PMC4329176

[5] Liu S Zheng R Zhang M Zhang S Sun X Chen W. Incidence and mortality of colorectal cancer in China, 2011. Chin J Cancer Res. (2015) 27:22–8. 10.3978/j.issn.1000-9604.2015.02.01 25717222 PMC4329182

[6] Biagi JJ Raphael MJ Mackillop WJ Kong W King WD Booth CM. Association between time to initiation of adjuvant chemotherapy and survival in colorectal cancer: a systematic review and meta-analysis. JAMA. (2011) 305:2335–42. 10.1001/jama.2011.749 21642686

[7] Duffy MJ Lamerz R Haglund C Nicolini A Kalousová M Holubec L. Tumor markers in colorectal cancer, gastric cancer and gastrointestinal stromal cancers: European group on tumor markers 2014 guidelines update. Int J Cancer. (2014) 134:2513–22. 10.1002/ijc.28384 23852704 PMC4217376

[8] Ye SB Cheng YK Zhang L Wang XP Wang L Lan P. Prognostic value of estrogen receptor-alpha and progesterone receptor in curatively resected colorectal cancer: a retrospective analysis with independent validations. BMC Cancer. (2019) 19:933. 10.1186/s12885-019-5918-4 31590647 PMC6781392

[9] Caiazza F Ryan EJ Doherty G Winter DC Sheahan K. Estrogen receptors and their implications in colorectal carcinogenesis. Front Oncol. (2015) 5:19. 10.3389/fonc.2015.00019 25699240 PMC4313613

[10] Nüssler NC Reinbacher K Shanny N Schirmeier A Glanemann M Neuhaus P. Sex-specific differences in the expression levels of estrogen receptor subtypes in colorectal cancer. Gend Med. (2008) 5:209–17. 10.1016/j.genm.2008.07.005 18727987

[11] Liu D. Gene signatures of estrogen and progesterone receptor pathways predict the prognosis of colorectal cancer. FEBS J. (2016) 283:3115–33. 10.1111/febs.13798 27376509

[12] Camp RL Dolled-Filhart M Rimm DL. X-tile: a new bio-informatics tool for biomarker assessment and outcome-based cut-point optimization. Clin Cancer Res. (2004) 10:7252–9. 10.1158/1078-0432.CCR-04-0713 15534099

[13] Simon RM Paik S Hayes DF. Use of archived specimens in evaluation of prognostic and predictive biomarkers. J Natl Cancer Inst. (2009) 101:1446–52. 10.1093/jnci/djp335 19815849 PMC2782246

[14] Chewchuk S Guo B Parissenti AM. Alterations in estrogen signalling pathways upon acquisition of anthracycline resistance in breast tumor cells. PLoS ONE. (2017) 12:e172244. 10.1371/journal.pone.0172244 28196134 PMC5308870

[15] Chang FW Fan HC Liu JM Fan TP Jing J Yang CL. Estrogen enhances the expression of the multidrug transporter gene abcg2-increasing drug resistance of breast cancer cells through estrogen receptors. Int J Mol Sci. (2017) 18:E163. 10.3390/ijms18010163 28098816 PMC5297796

[16] Aggarwal BB Sung B. NF-κB in cancer: a matter of life and death. Cancer Discov. (2011) 1:469–71. 10.1158/2159-8290.CD-11-0260 22586649 PMC3392037

[17] Özeş AR Miller DF Özeş ON Fang F Liu Y Matei D. NF-κB-HOTAIR axis links DNA damage response, chemoresistance and cellular senescence in ovarian cancer. Oncogene. (2016) 35:5350–61. 10.1038/onc.2016.75 27041570 PMC5050052

[18] Kuo WY Hwu L Wu CY Lee JS Chang CW Liu RS. STAT3/NF-κB-regulated lentiviral TK/GCV suicide gene therapy for Cisplatin-Resistant triple-negative breast cancer. Theranostics. (2017) 7:647–63. 10.7150/thno.16827 28255357 PMC5327640

[19] Chen MC Lee NH Hsu HH Ho TJ Tu CC Chen RJ. Inhibition of NF-κB and metastasis in irinotecan (CPT-11)-resistant LoVo colon cancer cells by thymoquinone via JNK and p38. Environ Toxicol. (2017) 32:669–78. 10.1002/tox.22268 27060453

[20] Schurmann R Cronin M Meyer JU. Estrogen plus progestin and colorectal cancer in postmenopausal women. N Engl J Med. (2004) 350:2417–9. 10.1056/NEJM200406033502319 15179699

[21] Green J Czanner G Reeves G Watson J Wise L Roddam A. Menopausal hormone therapy and risk of gastrointestinal cancer: nested case-control study within a prospective cohort, and meta-analysis. Int J Cancer. (2012) 130:2387–96. 10.1002/ijc.26236 21671473

[22] Marjoribanks J Farquhar C Roberts H Lethaby A Lee J. Long-term hormone therapy for perimenopausal and postmenopausal women. Cochrane Database Syst Rev. (2017) 1:D4143. 10.1002/14651858.CD004143.pub5 28093732 PMC6465148

[23] Sargent DJ Marsoni S Monges G Thibodeau SN Labianca R Hamilton SR. Defective mismatch repair as a predictive marker for lack of efficacy of fluorouracil-based adjuvant therapy in colon cancer. J Clin Oncol. (2010) 28:3219–26. 10.1200/JCO.2009.27.1825 20498393 PMC2903323

[24] Hanahan D Weinberg RA. Hallmarks of cancer: the next generation. Cell. (2011) 144:646–74. 10.1016/j.cell.2011.02.013 21376230

