# Supplementary material for: The Predictive Value of Estrogen Receptor 1 on Adjuvant Chemotherapy in Locally Advanced Colorectal Cancer: A Retrospective Analysis With Independent Validation and Its Potential Mechanism
Source: Front Oncol. 2020 Mar 20;10:214. doi: 10.3389/fonc.2020.00214 (PMC7100261; doi:10.3389/fonc.2020.00214)
Supplement: Supplementary file 1 [file Image_1.pdf]

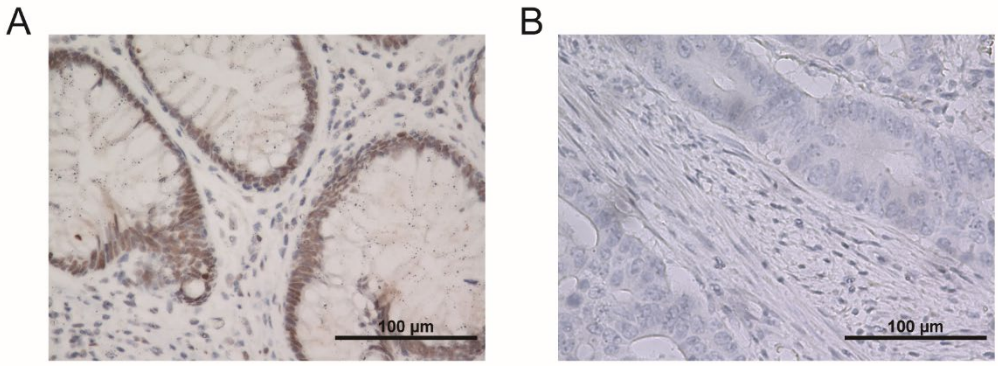

S Figure 1. Immunohistochemical staining for ESR1 in human colorectal cancer. Our data showed expression of ESR1 (A, X 400) and negative expression (B, X 400) in tumor tissues from patients with CRC.

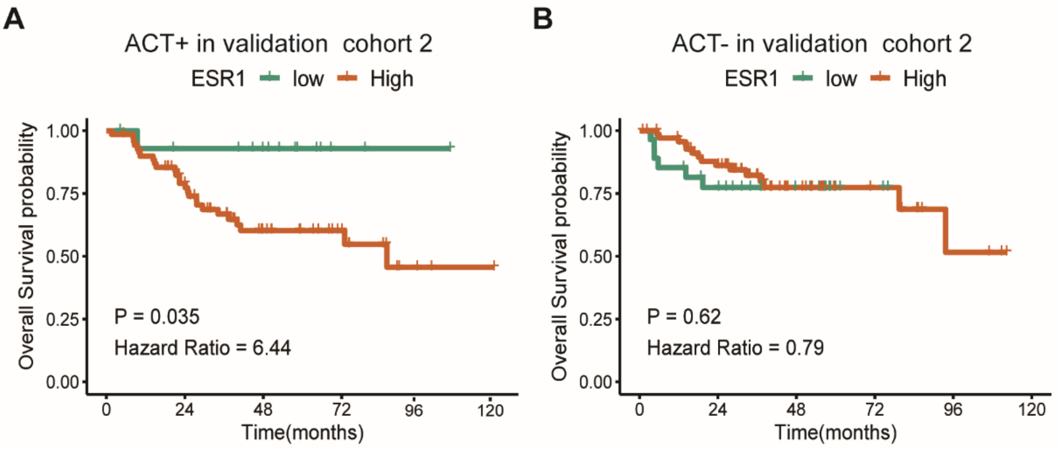

S Figure 2. Kaplan-Meier survival curves of DFS in CRC patients from external validation cohort 1 (**A**) with adjuvant chemotherapy and (**B**) without adjuvant chemotherapy by ESR1 status. Kaplan-Meier survival curves of DFS in CRC patients from external validation cohort 2 (**C**) with adjuvant chemotherapy and (**D**) without adjuvant chemotherapy by ESR1 status. Log-rank analysis was used to test for significance.
